# The FOXA transcription factors in allergic airway disease and asthma

**DOI:** 10.3389/falgy.2026.1770114

**Published:** 2026-03-05

**Authors:** Diana C. Yánez, Ching-In Lau, Jasmine Rowell, Eden Zhang, Tessa Crompton

**Affiliations:** UCL Great Ormond Street Institute of Child Health, London, United Kingdom

**Keywords:** allergic airway disease, asthma, FOXA, goblet cells, T-cells, Th2, tissue remodelling

## Abstract

The Forkhead box A (FOXA) transcription factors are pioneer factors that orchestrate diverse cellular and developmental processes. Within the respiratory system, FOXA1, FOXA2, and FOXA3 have emerged as modulators of pulmonary inflammation, with important implications for the pathogenesis of allergic asthma. In healthy lung and airways, FOXA1 and FOXA2 maintain epithelial integrity and promote ciliated cell development, thereby preserving homeostasis. FOXA2 additionally suppresses Th2 differentiation and goblet cell metaplasia and mucus overproduction. During allergic airway inflammation, FOXA2 expression is decreased and FOXA3 expression is induced by Th2 cytokines. Dysregulation of the FOXA regulatory network disrupts epithelial cell homeostasis and amplifies allergic inflammation, contributing to airway remodelling. Here we will review the potential mechanisms through which the FOXA family modulates airway inflammation, while also addressing gaps in current understanding, such as the context specific regulation of FOXA isoforms, their interplay with cytokine signalling pathways, and the translational relevance of these findings to therapeutic strategies in asthma.

## Introduction

Asthma is an heterogeneous chronic airway inflammatory disease characterized by variable airflow limitation associated with cough, wheezing, shortness-of-breath and chest tightness (1). It is one of the most common non-communicable respiratory conditions affecting over 300 million people worldwide, with highest prevalence in high-income countries (2).

Asthma can be broadly subdivided into two major endotypes: Type 2-high and Type 2-low asthma (3). Type 2-high asthma is orchestrated by Th2 cytokines [Interleukin (IL)-4, IL-5, and IL-13], which are produced by T-helper 2 (TH2) T-cells and innate lymphoid type 2 (ILC2) cells. This endotype is characterized by excessive production of allergen-specific IgE, airway eosinophilia and atopy (4). In contrast, Type 2-low asthma patients lack hallmark features of type-2 inflammation but exhibit neutrophilic or paucigranulocytic airway inflammation, driven mainly by Th1- and Th17-mediated pathways (3, 5).

Allergic asthma (type 2-high) accounts for more than half of asthma cases, and is triggered by inhalation of environmental stimuli such as pollens, house dust mites (HDMs), chemicals or mould spores, leading to eosinophilic airway infiltration, chronic airway inflammation, mucus hyperproduction, and airway hyperresponsiveness (2). Current understanding indicates that TH2 cells drive allergic inflammation by interacting with innate immune cells and airway epithelial cells to release a plethora of TH2 cytokines.

During the sensitization phase, inhaled allergens are endocytosed by antigen presenting cells, most commonly dendritic cells (DC), which present antigen to naïve CD4+ T-cells to prime an antigen-specific effector T-cell response (6). This leads to activation and polarization of TH2 and ILC2 cells, which secrete Th2-cytokines IL-4, IL-5, and IL-13 that orchestrate the hallmark features of asthma (3, 7).

IL-4 plays a central role in induction of IgE production by B cells. The secreted IgE then binds to high-affinity Fc*ε*RI receptors on mast cells and basophils. Upon allergen re-exposure, antigen cross-linking of membrane-bound IgE triggers rapid activation of these cells, leading to release of inflammatory mediators such as histamine and leukotrienes (8, 9).

The airway epithelium includes basal cells, ciliated cells, goblet cells, and club cells. In asthma, structural change of the airway epithelium involves increases in goblet cell numbers, with upregulation of mucin gene expression (such as *MUC5AC*), with associated mucus overproduction. This can lead to mucus plugging and airway obstruction, and potentially fatal asthma exacerbations (10, 11). Growing evidence indicates that Th2 cytokines upregulate mucin gene expression in goblet cells, playing an important role in airway goblet cell metaplasia and hyperplasia (12). Expression of mucin genes *MUC2*, *MUC4* and *MUC5AC* were increased in asthma patients, while *MUC5B* was significantly decreased (11). Overexpression of MUC5AC is observed in airway inflammation and its upregulation in goblet cells is a key pathological feature of asthma (13, 14).

Goblet-cell expansion occurs through both hyperplasia (increased cell number) and metaplasia, the latter arising when club cells or ciliated cells transdifferentiate into goblet cells (15). The transcription factor SPDEF is strongly induced after allergen exposure or Th2 cytokine stimulation and drives goblet cell differentiation and metaplasia (16, 17). Club cells secrete the anti-inflammatory and epithelial-protective protein SCGB1A1. Reduced SCGB1A1 expression correlates with persistent airway inflammation and lung-function decline (18, 19). Asthma patients have significantly lower SCGB1A1 levels in serum and airways compared with healthy controls (18, 19).

In addition, the airway epithelium actively drives asthma pathophysiology by releasing epithelial cytokines, alarmins, such as thymic stromal lymphopoietin (TSLP), IL-33, and IL-25, which activate both innate and adaptive immune cells, including TH2 and ILC2 cells, eosinophils and mast cells, leading to elevated production of IL-4, IL-5 and IL-13, and airway eosinophilia, IgE production, mucus hypersecretion, and airway hyperresponsiveness (20, 21).

Persistent inflammation in asthma leads to chronic tissue injury and airway remodelling. This process encompasses epithelial dysfunction, smooth muscle hypertrophy, increased vascularity, fibroblast activation, and enhanced expression of angiogenic and fibrogenic mediators. The resulting extracellular matrix deposition and structural alterations progressively impair lung function (22).

TH2 cytokines play a central role in driving chronic airway inflammation, excessive mucus production, and the development of fibrosis, all of which collectively exacerbate airway remodelling. IL-9 (secreted by T-cells, ILC2 and activated Mast cells) IL-4, and IL-13 induce goblet cell hyperplasia/metaplasia, airway hyperreactivity and mucus over-production (23–31). IL-5 is responsible for activation and migration of eosinophils, thereby sustaining eosinophilic inflammation, with presence of intraepithelial eosinophils, while elevated eosinophils in the bronchial submucosa cause epithelial damage (32–35). Subsequently, eosinophil-derived TGFβ promotes smooth muscle cell hyperplasia and fibroblast proliferation (36, 37). Vascular endothelial growth factor (VEGF) further contributes to airway remodelling by promoting angiogenesis and increased vascular hyperpermeability (38, 39). Together, these mediators establish a persistent remodelling environment in asthma, where chronic inflammation, structural cell activation and dysregulated repair processes progressively alter airway architecture and function.

Type-2 asthma is therefore a complex disorder that is not triggered by one factor or mechanism, but rather by a combination of factors, involving interaction of environmental triggers, immune-mediated effects, and defects in airway epithelium. Here we review the multiple roles of the Forkhead box (FOX) transcription factors FOXA1–3 in allergic asthma and discuss their potential as therapeutic targets.

## The FOXA family of transcription factors

The transcription factors FOXA1–3, are pioneer factors with the ability to bind compacted chromatin and remodel its accessibility, thereby establishing epigenetic landscapes that direct lineage specification (40). Foxa1 shares 95% DNA sequence identity and Foxa2 shares 90% with Foxa3 within the forkhead domain (41). Outside this domain, Foxa1 and Foxa2 share higher similarity with one another (over 30%) than Foxa3. During mouse embryonic development, Foxa2 is first expressed at embryonic day (E) 6.5 in the notochord and the primitive streak, followed by Foxa1 at E7 in the notochord, midbrain floorplate and endoderm, and Foxa3 at E8.5 in foregut endoderm organs (42).

These evolutionarily conserved DNA-binding regulators play indispensable roles in organogenesis, particularly in endoderm-derived tissues such as liver, pancreas, and lung (43). By priming enhancers and facilitating recruitment of additional transcriptional machinery, Foxa factors orchestrate the activation of developmental gene networks essential for embryonic differentiation, and tissue identity/homeostasis in adults (42, 44–46).

The Foxa family are important for T-cell development and homeostasis. Foxa1 and Foxa2 are expressed in thymic epithelial cells (TEC), regulating their development and function. Conditional deletion of both genes from TEC led to fewer thymocytes, but more CD4 regulatory T cells (Treg) cells (47). FOXA1 and FOXA2 are also expressed in thymocytes, where they regulate RNA splicing during T-cell development, affecting the peripheral CD4 T cell pool (48, 49). FOXA2 has a role in the homeostatic balance of Th1/Th2 cell differentiation (50), whereas FOXA1 expression in a subset of Treg drives its differentiation and suppressive function (51).

The dual roles of FOXA proteins in initiating chromatin accessibility and sustaining transcriptional programs underscore their importance to development and homeostasis.

The FOXA transcription factors are implicated in inflammatory airway disorders including chronic obstructive pulmonary disease (COPD), pulmonary fibrosis, emphysema and asthma in human and mouse (52–63). In addition, genome wide association studies (GWAS) and meta analyses have linked respiratory disease, asthma and asthma subtypes to variants of *FOXA3* (62, 64, 65), including later-onset asthma, non-atopic asthma, asthma associated with obesity; and asthma associated with endometriosis (61, 63). Furthermore, GWAS has associated both *FOXA1* and *FOXA3* with blood eosinophil count (66, 67).

## FOXA in asthma

### FOXA2

Foxa2 is essential for lung morphogenesis and is detected in mouse lung embryonic development from E10, predominantly in bronchial and bronchiolar epithelial cells. FOXA2 is co-expressed with thyroid transcription factor 1 (TTF-1), Clara cell secretary protein (CCSP), and surfactant proteins, important for epithelial cell differentiation and production of surfactant (68). FOXA2 regulates expression of genes involved in surfactant protein and lipid synthesis, host defence, and antioxidant production and is important in the transition to air breathing at birth (69). After birth, FOXA2 is also required for postnatal alveolarization and epithelial cell differentiation.

### FOXA2 function in airway epithelial cells

Conditional deletion of *Foxa2* from respiratory epithelial cells leads to abnormalities in alveolarization, goblet cell hyperplasia, spontaneous cellular infiltration and mucus overproduction (70). This also results in airspace enlargement and spontaneous inflammation, associated with recruitment and activation of myeloid DC, infiltration of Th2 cells and increased levels of Th2 cytokines and chemokines during postnatal lung development (71). All these abnormalities are hallmarks of several inflammatory conditions, including asthma. Multiple lines of evidence demonstrate that Foxa2 plays a pivotal role in allergic airway disease by predominately regulating goblet cell hyperplasia and mucus production. Immunostaining of lung epithelia sections from children with chronic lung disease showed a lack of FOXA2 staining in mucus-producing cells. In support of this, FOXA2 expression is reduced in airways of mild/moderate asthmatic patients compared to healthy controls, whereas, expression of mucin genes *MUC5AC* and *CLCA1* are negatively correlated to FOXA2 expression (54). Low levels of FOXA2 were seen in goblet cells of mouse lung epithelia after administration of IL-4, IL-13, and OVA, but its expression was maintained in goblet cell precursors (70). Likewise, suppression of *Foxa2* in mouse lung epithelia caused spontaneous goblet cell hyperplasia and increased expression of the mucin gene *Muc5ac* (70). FOXA2 can suppress goblet cell metaplasia and mucus production by counteracting the effects of IL-13/STAT6 signalling, which via their downstream effector SPDEF, increase expression of *Muc5ac* (72).

SPDEF plays a central role in the regulation of goblet cell differentiation and mucin biosynthesis in epithelial cells in the lung. Conditional ectopic expression of SPDEF in Clara cells inhibited expression of *Foxa2* and genes it regulates, such as *Titf1, Sftpa, Sftpb, Sftpd, Scgb1a1* after OVA sensitization (73). However, it is not clear whether the effects of SPDEF expression are direct or are indirect effects because of the downregulation of FOXA2.

FOXA2 functions as an upstream activator of SCGB1A1, a Clara cell-derived protein with anti-inflammatory properties connected with air barrier integrity in asthma (19, 74). Reduction in *SCGB1A1* and *FOXA2* transcripts have been observed in bronchoalveolar lavage fluid and peripheral blood from asthmatic patients relative to healthy controls. Interesting, overexpression of FOXA2 in epithelial cells exposed to Th2 cytokines or rhinovirus led to recovery of SCGB1A1 expression (19). FOXA2 also protects against bacterial lung infection. Its inactivation by pathogens or inflammatory pathways leads to impaired mucus control, weakened antimicrobial defences, and heightened allergic airway inflammation (72, 75–77).

Higher recruitment and activation of myeloid DC and higher expression of chemoattractants for Th2 cells were seen postnatally on conditional deletion of *Foxa2* from airway epithelial cells (71). However, the mechanisms underlying recruitment and activation of pulmonary DC after *Foxa2*-deletion are unknown.

FOXA2 also regulates leukotrienes (LT) and their related enzymes, important inflammatory mediators of Th2-dependent pulmonary inflammation. Conditional deletion of *Foxa2* from airway epithelium activated the LT signalling pathway and increased expression of Th2 cytokines (78).

### FOXA2 in T-cells

FOXA2 is expressed in T-cells, with higher expression in Th2 cells than Th1 cells, and contributes to allergic airway inflammation by modulating T helper differentiation (48, 50). Conditional deletion of *Foxa2* from T-cells suppressed Th1 and enhanced Th2 differentiation *in vitro*, with higher expression of the Th2 transcription factor GATA3 and Th2 cytokines IL-4 and IL-13, and lower expression of Th1 transcription factor TBET and IFNγ. Furthermore, conditional deletion of *Foxa2* from T-cells increased infiltration of innate inflammatory cells, with Th2 cytokine secretion, mucus over-production, and higher expression of *Muc5ac* and its transcriptional activator *Spdef* after allergen-challenge in mouse allergic airway inflammation (50). This indicates that FOXA2's involvement in Th2-mediated disease is complex, as it is not only important in the airway epithelial cells but also in T-cells to maintain the balance of T-cell differentiation/function and lung homeostasis.

### FOXA1

FOXA1 is expressed in the trachea, bronchioles, and type-2 alveolar cells (79). It contributes to the regulation of epithelial differentiation and morphogenesis during lung development alongside FOXA2. Although FOXA1 alone is not essential for lung formation, it can partially compensate for absence of FOXA2, whereas combined deletion of *Foxa1* and *Foxa2* results in severe defects in lung morphogenesis, indicating some functional redundancy (69, 70).

Chromatin immunoprecipitation and RNA sequencing have identified direct transcriptional targets of *FOXA1* in human bronchial epithelial (HBE) cells, which included *SPDEF* (80). FOXA1 was also shown to regulate expression of collagen family members, periostin, matrix metallopeptidase 9 and 10, laminin subunit α-4 and keratin-associated genes and shared many binding targets with FOXA2. Overall, FOXA1 was shown to regulate cell adhesion, maintenance of transepithelial resistance, and epithelial identity in healthy HBE, where it also represses *FOXA2*, indicating a complex interplay between overlapping/compensatory functions with FOXA2, and repression of FOXA2 expression.

During airway inflammation, FOXA1 participates in the transcriptional network that maintains epithelial identity and coordinates responses to inflammatory stimuli, indirectly influencing mucus production and epithelial remodelling, by regulating the epithelial transcriptional program (80). Since, FOXA1 and FOXA2 are co-expressed in respiratory epithelial cells, increased expression of FOXA1 during airway inflammation may partially compensate for deletion of FOXA2. FOXA1 works in tandem with FOXA2, and while FOXA2 strongly suppresses goblet cell metaplasia, FOXA1 provides supportive regulation (80, 81).

### FOXA3

After birth, FOXA3 is highly expressed in liver, pancreas and intestine, but detected at low levels in the respiratory tract (82, 83). Interestingly, FOXA3 is highly expressed in Clara/goblet cells localized in upper and lower airway epithelium from patients with asthma and COPD (52, 65, 73, 84). Transcriptome-wide association study analysis has identified FOXA3 as a regulator of mucus production in nasal airway epithelium in children with asthma (85). Integration of cell type deconvolution and expression quantitative trait loci analysis showed that FOXA3 is associated with asthma and expressed in secretory cells in upper and lower airway epithelium (65, 84).

*FOXA3* expression correlates with *CLCA1* expression, an IL-13/Th2-inducible gene upregulated in asthma (54, 86, 87). In human lung epithelial cells *in vitro* FOXA3 expression is significantly increased after IL-13 administration, and in mice after allergen challenge FOXA3 induces expression of *Tslp* and *Ccl26*, which contribute to amplification of Th2-mediated immune responses (60, 88). Moreover, conditional ectopic expression of FOXA3 in non-ciliated airway epithelial cells in neonates caused spontaneous goblet cell metaplasia, and pulmonary cellular infiltration of eosinophils, ILCs, T-cells and DC with higher Th2 cytokine mRNA expression (17).

**Figure 1:**
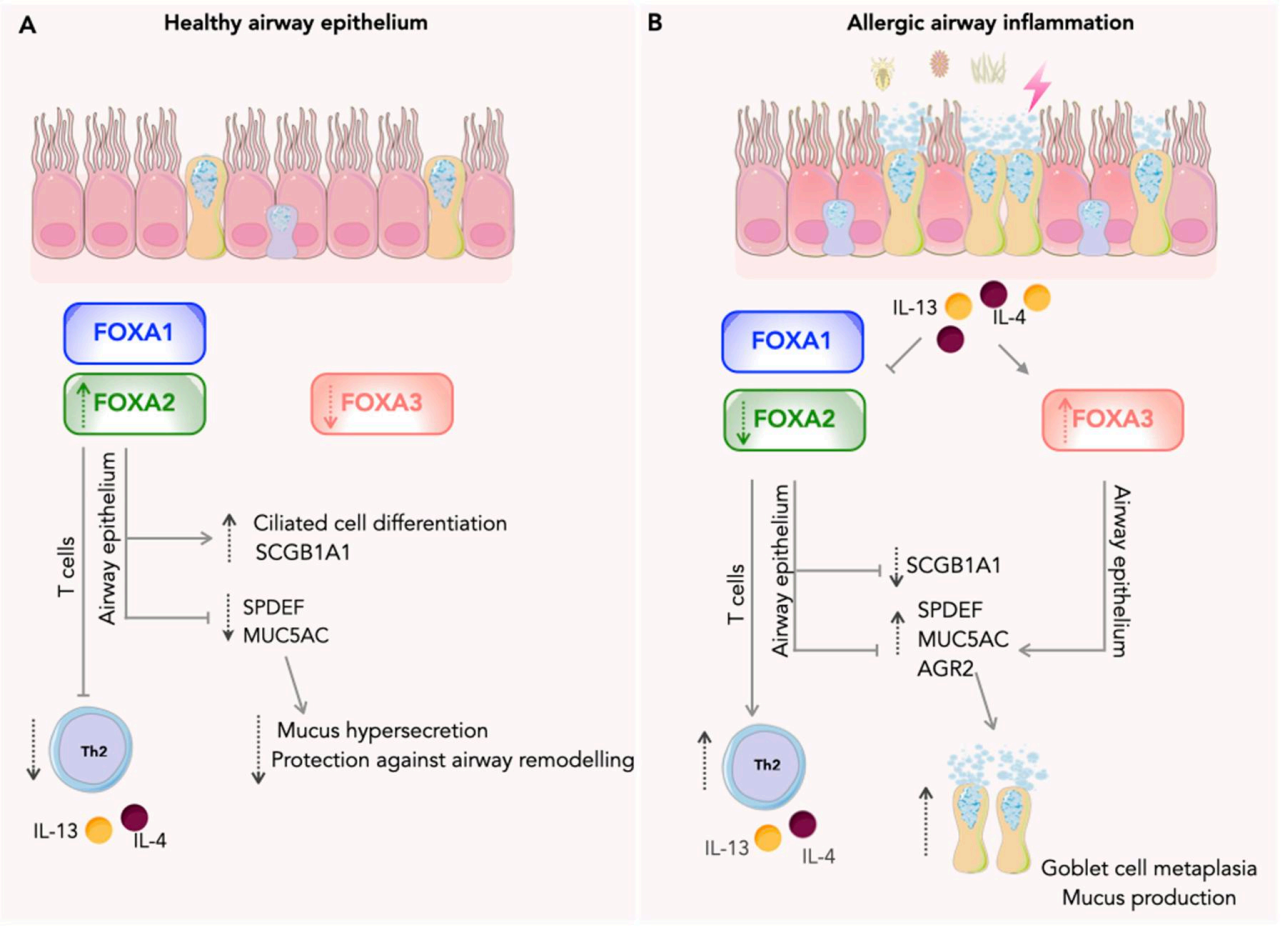
The distinct roles of the FOXA transcription factors in airway epithelial regulation and inflammation. Graphical abstract illustrating the distinct roles of FOXA transcription factors in airway epithelial regulation and inflammation. **(A)** Under healthy conditions, FOXA1 and FOXA2 preserve epithelial integrity and drive ciliated cell differentiation, thereby maintaining mucus homeostasis. FOXA2 also sustains expression of the Clara cell protein SCGB1A1, a key airway defence molecule. **(B)** During allergic airway inflammation or viral infection, FOXA2 is down-regulated, leading to increased expression of *SPDEF, MUC5AC and ARG2,* genes involved in goblet metaplasia and mucus overproduction. Deletion of FOXA2 from T cells promotes Th2 differentiation and amplifies Th2-mediated inflammation. FOXA3 becomes up-regulated in epithelial cells during asthma, activating mucin gene expression and driving goblet cell metaplasia with excessive mucus secretion. These molecular events contribute to the classical features of asthma, including airway remodelling characterised by goblet cell metaplasia, mucus hyper-secretion, and impaired epithelial barrier function. FOXA, forkhead Box A; SCGB1A1, secretoglobin family 1A Member 1; ARG2, Arginase, Type II. SPDEF, SAM pointed domain containing ETS transcription factor; MUC5AC, Mucin 5AC; IL-13 Interleukin-13; IL-4, Interleukin-4; Th2, T helper cell type 2. Pink cells represent airway epithelial cells and yellow cells represent goblet cells. T cells are shown in blue.

FOXA3 induces expression of *Muc5ac* and *Agr2*, promoting mucus cell metaplasia and mucus production in response to Th2 cytokines or virus infection (17, 52). SPDEF induces expression of FOXA3 and AGR2 in goblet cells *in vivo*, suggesting cooperative regulation of gene expression during goblet cell differentiation (73); conversely, FOXA3 also promotes SPDEF expression, yet its effects on mucus production genes, including *AGR2* and *MUC5AC*, are not entirely dependent on SPDEF, highlighting a complex regulatory network in which FOXA3 can function independently for certain processes (52). It is plausible that SPDEF and FOXA3 engage in a reciprocal regulatory loop, wherein each factor enhances the expression of the other, thereby reinforcing a transcriptional program that drives the differentiation of mucus-secreting goblet cells, but this requires further investigation. However, in *Foxa3*-/- mice after allergen-challenge, no effect on mucus cell metaplasia or *Muc5ac* mRNA levels were seen compared to controls, despite higher serum IgE and eosinophils (54). Taken together, these data suggest that FOXA3 plays a role in secretory cell function during Th2 airway inflammation. Figure 1 summarizes the key functions of FOXA1, FOXA2, and FOXA3 under both inflammatory and healthy airway conditions.

Viral infection and IFN-β exposure of primary human bronchial epithelial cells *in vitro* increased *FOXA3*, *SPDEF* and *MUC5AC* expression. Interesting, FOXA3-overexpression in airway epithelial cells inhibited Rhinovirus clearance and reduced expression of antiviral genes, including interferon-stimulated genes (ISGs) *in vitro* and *in vivo* (52). Thus, FOXA3 can weaken epithelial defence against viral infections while driving mucus-producing goblet cell differentiation, creating a permissive environment for viral persistence during airway inflammation.

## Discussion

The FOXA transcription factors have potential as therapeutic targets in asthma. FOXA2 has been consistently highlighted as a protective regulator, safeguarding epithelial stability and limiting goblet cell metaplasia by repressing Th2 differentiation and SPDEF and mucin gene programs. Experimental models show that restoring or enhancing FOXA2 activity can dampen Th2-mediated inflammation and mucus overproduction, pointing to its value as a candidate for intervention. FOXA1, though less directly studied in asthma, appears to function as a chromatin-priming factor that influences epithelial lineage decisions, and possibly supports FOXA2's protective effects. Small molecule inhibitors have been identified that target the DNA binding domains of FOXA1/2/3 (89), but small molecules that directly enhance FOXA2 expression or activity are not to our knowledge available. However, FOXA2 occupancy at its DNA binding sites is greatly increased by agonist binding to type II nuclear receptors FXR and LXRα in liver (90). This raises the possibility that FXR/LXRα agonism could be used to increase FOXA2 activity in lung.

In contrast, FOXA3 is upregulated in response to Th2 cytokines or virus and acts to intensify mucus secretion, suggesting that strategies aimed at inhibiting FOXA3 may prevent airway remodelling. The pharmacological compound magnolol is widely used in Chinese traditional medicine and has been shown to specifically inhibit *FOXA3* transcription, while not affecting *FOXA1/2* expression (91). Magnolol may have therapeutic value in asthma, with appropriate targeting to the airways, to avoid potential off-target toxicity.

Despite these promising insights, several uncertainties remain. It is not yet clear whether FOXA2 directly antagonizes FOXA3 activity or instead acts through shared downstream pathways such as SPDEF. Moreover, findings from murine models require validation in diverse human asthma subtypes, including those not driven by Th2 inflammation. Finally, systemic manipulation of FOXA factors raises safety concerns given their broad involvement in metabolic and developmental processes. Future research should focus on isoform-specific and cell-targeted approaches, complemented by longitudinal human studies to evaluate FOXA activity as a biomarker for disease progression and therapeutic responses.

Different pathways have been related with function of FOXA2 in allergic inflammation. Adequate FOXA2 activity is critical for promoting ciliated cell differentiation, suppressing goblet cell metaplasia, and limiting mucus hypersecretion, functions that collectively protect against Th2-driven airway inflammation. The long noncoding RNA Falcor acts in cis to regulate *Foxa2* transcription, thereby maintaining epithelial homeostasis in the lung. Loss of Falcor reduces FOXA2 expression, resulting in impaired epithelial repair following injury and increased susceptibility to inflammatory responses (92). Their combined regulatory role seems important for sustaining epithelial integrity under steady-state conditions and for enabling effective regeneration after allergen-induced damage, underscoring FOXA2's importance in the pathogenesis of asthma.

The Hedgehog (Hh) signalling pathway regulates lung development and homeostasis and is dysregulated in inflammatory lung conditions, such as asthma and COPD (93). Hh signalling and the FOXA transcription factors interact in many tissues, and FOXA2 is a direct target of Shh signalling, while FOXA1 can regulate Hh pathway activation (49, 94–97). In airway disease, FOXA2 and Hh signalling have opposing functions, as FOXA2 suppresses pathways that Hh activates (93, 98–105). Inhibition of Hh pathway activation is under investigation as a therapeutic approach for treatment of asthma (98, 99, 106, 107), but the impact of Hh-inhibition on FOXA expression in airway epithelial cells is unknown. Current evidence suggests that loss of FOXA2 is a permissive factor for Hh-driven airway remodelling, but whether FOXA2 directly represses Hh components (e.g., Shh, Gli1) remains unresolved.

FOXA3 compromises epithelial antiviral defences while simultaneously promoting differentiation of mucus-secreting goblet cells and modulating Th1/Th2 responses. Mechanistically, FOXA3 suppresses ISG expression, thereby attenuating innate antiviral immunity, and leading to upregulation of Th2-related genes in the lung epithelium, while driving goblet cell metaplasia and mucus hypersecretion that impair mucociliary clearance. This dual activity establishes a permissive environment for viral persistence and amplifies allergic airway inflammation.

Taken together, current evidence indicates that the FOXA transcription factors play an important role in the pathogenesis of allergic asthma. FOXA2 expression protects against disease severity, whereas upregulation of FOXA3 is involved in driving allergic inflammation. The FOXA family may therefore good candidate therapeutic targets for future investigation.
